# Drug-Induced Thrombocytopenia: A Case Involving Vancomycin

**DOI:** 10.7759/cureus.41874

**Published:** 2023-07-14

**Authors:** Alexander Barnecet Pérez, Caleb A Niehues, Courtney M Hicks, Hemal Patel, Dayan Sanchez, David E Martin, Suporn Sukpraprut-Braaten

**Affiliations:** 1 Internal Medicine, Unity Health, Searcy, USA; 2 Graduate Medical Education, Unity Health, Searcy, USA; 3 Infectious Disease, Unity Health, Searcy, USA

**Keywords:** drug discontinuation, immune-mediated thrombocytopenia, vancomycin infusion, drug-induced thrombocytopenia, vancomycin-induced immune thrombocytopenia

## Abstract

Drug-induced thrombocytopenia (DIT) is a rare adverse effect that occurs when administering various medications. The medications associated with this possible adverse effect include heparin, penicillin, furosemide, vancomycin, non-steroidal anti-inflammatory drugs, ranitidine, and many others. DIT causes a rapid decrease in platelet counts after drug administration and typically resolves once the offending agent has been discontinued. The induced thrombocytopenia increases the bleeding risk and possibility of adverse effects throughout a hospital course. In this case report, we look at the presenting symptoms and treatment course of an interesting case of DIT that occurred following the administration of vancomycin.

## Introduction

Drug-induced thrombocytopenia (DIT) is an uncommon adverse effect of various medications, including heparin, vancomycin, penicillin, furosemide, non-steroidal anti-inflammatory drugs, ranitidine, and many others [1]. This complication presents as a markedly decreased platelet count following medication administration [1]. The current understanding of the development of this adverse effect includes various mechanisms, with the most common being the development of autoantibodies that specifically target the individual’s platelets [2,3]. The resulting thrombocytopenia associated with the platelet destruction in DIT can result in platelet levels below 50 × 10^3^/μL, increasing the individual’s overall bleeding risk [1]. Current initial treatment practices for DIT include discontinuation of the offending medication with additional platelet transfusions, intravenous immunoglobulin (IVIG), and corticosteroid administration for life-threatening bleeding.

The following case presents a patient who experienced DIT following the administration of vancomycin. It is known that the common adverse effects associated with vancomycin include phlebitis, nephrotoxicity, ototoxicity, hypersensitivity reactions, chills, exanthem, and fever [4]. Due to the frequent use of vancomycin in the hospital setting and the uncommon presentation of vancomycin-induced thrombocytopenia, it is important to increase physician awareness and knowledge in managing this potentially life-threatening condition.

## Case presentation

An 80-year-old Caucasian female presented to the Emergency Department after experiencing a fall at home resulting in a distal left supracondylar periprosthetic femur fracture that was repaired with an open reduction internal fixation (ORIF) with less invasive stabilization system (LISS). She had a past medical history of coronary artery disease, stage 4 chronic kidney disease, essential hypertension, and osteoporosis. After successful surgical fixation and appropriate progression thereafter, the patient was discharged to a rehabilitation facility. She returned to the hospital 14 days later due to concern of possibly developing a surgical site wound infection. At that time, she presented with erythema, warmth, and purulent drainage from the surgical incision site. She was readmitted to the hospital and started on vancomycin (day 0). The patient underwent an incision and drainage where purulent drainage was found. Wound cultures were collected from the purulent drainage and were found to have no growth. Due to the purulent drainage, vancomycin was continued for treatment of the surgical site incision infection. The patient was once again stabilized and discharged to a rehabilitation facility with a plan to complete a six-week course of vancomycin.

During the initial lab work of the visit with concern for surgical site wound infection, her platelet count was found to be normal at 335×10^3^/μL. Platelet counts obtained from the daily lab work remained between 200 and 300×10^3^/μL until day 6 following the initiation of vancomycin when platelets dropped to 195×10^3^/μL. On the day of discharge to the rehabilitation facility following incision and drainage, on day 7, the platelet count decreased to 57×10^3^/μL.

Seven days after discharge to the rehabilitation facility with the plan of completing a six-week course of vancomycin, the patient returned to the hospital, on day 14, due to thrombocytopenia that was found at the rehabilitation facility. While at the facility her platelets were found to be as low as 0×10^3^/μL on complete blood count (CBC) with auto-differential two days prior to reappearance to the hospital. When this platelet count was found, the patient received 2 units of platelets; however, her platelets count remained at 0×10^3^/μL on CBC with auto-differential. She denied hematemesis, hematuria, melena, hematochezia, or other bleeding/clotting disorders at the time of this presentation. The patient was also afebrile at the time of readmission, without chills, or night sweats. Hematology-oncology was consulted due to the thrombocytopenia, who suggested that Vancomycin MAY be the cause of the thrombocytopenia, which started to develop seven days following the initial dose.

**Figure 1:**
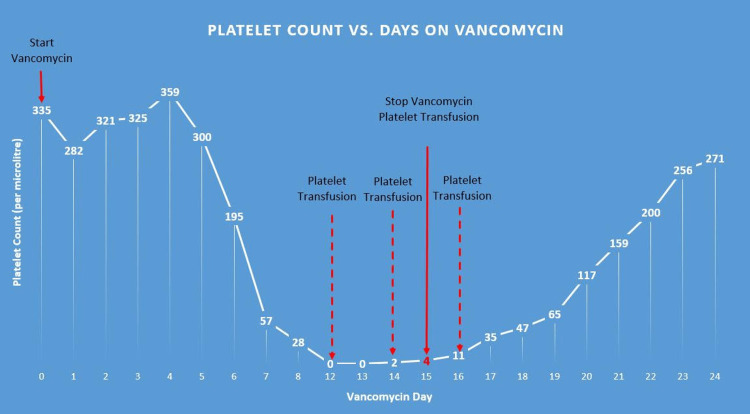
Platelet count and days on vancomycin

Initial lab work of the hospital admission in regard to thrombocytopenia found her platelet count to be 2×10^3^/μL. The patient was given Solu-Medrol 125 mg and a platelet transfusion on this day with a subsequent laboratory platelet count of 4×10^3^/μL the following day. Due to the concern of the vancomycin causing the thrombocytopenia, vancomycin was discontinued. Following the discontinuation of vancomycin, the infectious disease service was consulted for assistance with the antibiotic regimen and recommended transitioning the individual to ceftriaxone 2 g IV once a day. After the discontinuation and changing of antibiotics, the patient’s platelet levels began to trend upward toward normal slowly over the following six days. On day 21, the platelet count was found to be increased to 159×10^3^/μL, and as time progressed, it continued to trend upwards (Figure 1).

Figure 1 is a visualization reference of the platelet count trend that was experienced by the patient in this case. Upon admission on day 0, the patient received vancomycin due to the development of a surgical site infection. On day 7, the patient experienced a dramatic decrease in her overall platelet count to 57×10^3^/μL. On day 12, the platelet count dropped to 0×10^3^/μL. The patient received platelet transfusions on days 12 and 14, resulting in no significant improvement in platelet levels. The platelet count remained at 0 for two consecutive days. On day 15, vancomycin was stopped. Following vancomycin discontinuation, the platelet count increased to the normal range (above 150×10^3^/μL) on day 21.

Table 1 summarizes the highlights of the case.

**Table 1:** Review of the drug-induced thrombocytopenia case

Age and Gender	Source of Infection	Comorbidity	Lowest Platelet Count	Bleeding Symptoms	Treatment and Duration of Recovery	Recovery Time (Platelet Count >150 x10^9^)
An 80-year-old female	Surgical incision site	Coronary artery disease, type 2 diabetes mellitus, hypertension, stage 4 chronic kidney disease, rheumatoid arthritis, osteoporosis, and hyperlipidemia	0 x 10^3^/µL	None	Vancomycin was discontinued. Platelet infusions and Solu-Medrol were given	6 days

## Discussion

This case involves an 80-year-old female presenting on post-operative day 14 due to a surgical incision site infection following an ORIF with LISS procedure that was completed to repair a distal left supracondylar periprosthetic femur fracture, which was subsequently treated with vancomycin. She was then found to develop asymptomatic thrombocytopenia that resolved following the discontinuation of vancomycin despite efforts to treat it with steroids and platelet transfusions. This case involves an interesting adverse effect that is a rare occurrence with the use of vancomycin, with a reported occurrence of 7.1% in individuals being treated with vancomycin [5]. Using this case and other case reports from similar presentations, we are able to discuss and educate on the typical presentations of this adverse effect and the treatment course with the best outcomes [1,2,6-11].

Vancomycin is widely used in hospitals for the treatment of severe infections and has well-known side effects, including phlebitis, nephrotoxicity, ototoxicity, hypersensitivity reactions, red man syndrome, and neutropenia [4]. Vancomycin is not commonly thought of as an agent that causes DIT, as seen in this report, although, as presented here, it has been found to be a severe adverse effect that could lead to prolonged hospitalization and put the patient at a significantly increased risk for spontaneous bleeding. The mechanism that this adverse effect arises by following the administration of vancomycin is with the production of autoantibodies that specifically target the GPIIbIIIa or GPIbIX receptors on platelets and subsequently lead to the development of significant thrombocytopenia [3]. Furthermore, some autoantibodies bind to receptors on megakaryocytes, thus further impairing normal platelet production [12]. It is important to note that not all patients will present with signs of spontaneous bleeding, which would make the thrombocytopenia reported on daily lab work the only warning sign of this condition.

As discussed previously, the typical course of treatment for DIT includes the discontinuation of the presumptive offending medication, vancomycin in this example, along with the administration of platelet transfusions, IVIG, and/or steroids for life-threatening bleeding or severe thrombocytopenia. Although platelet transfusions and steroids were given to the patient in this case, significant improvement in platelet count was not seen until the discontinuation of vancomycin. This finding was compared to other case reports of vancomycin-induced thrombocytopenia, and it was consistently discovered that significant platelet recovery began following vancomycin discontinuation [2,5-14]. The normal time frame for platelet counts to return to levels greater than 150x10^3^/μL occurred between 5 and 7 days after vancomycin discontinuation. This correlates with the average lifespan length of a platelet, which is 7-10 days [15].

After analysis of this case report, we can conclude that physicians need to be made aware of the possible DIT associated with the administration of vancomycin. This adverse effect will be seen in patients experiencing progressive thrombocytopenia after receiving vancomycin for antibiotic treatment. It is essential to understand that the most crucial step in treatment is to stop the offending agent. Other adjuncts to treatment include giving platelet transfusions, steroids, and IVIG, following the discontinuation of vancomycin. If the exact cause of the thrombocytopenia is in question, then using the Adverse Drug Reaction Probability Scale, also known as Naranjo’s scale [16], could be a helpful tool in deciding if discontinuation of a medication would be of benefit. We can look at this case and see that the patient had a “probable” result on this scale that would have aided in the decision of antibiotic discontinuation. Early recognition and discontinuation of medicine will help decrease the risk of experiencing adverse effects, shorten hospital courses, and likely improve overall patient outcomes in individuals experiencing thrombocytopenia secondary to vancomycin.

## Conclusions

Vancomycin-induced thrombocytopenia is not a widely recognized complication of vancomycin administration. This adverse effect can be recognized as a development of thrombocytopenia shortly after the administration of vancomycin and may or may not present with bleeding symptoms. Under-recognition of this condition can affect a patient’s prognosis by increasing their overall bleeding risk. Therefore, noting instances of thrombocytopenia from vancomycin administration is prudent to raise awareness for earlier detection and to improve clinical outcomes. It is imperative to discontinue the administration of vancomycin if the patient develops thrombocytopenia due to the medication.
